# Aberrant cytokine pattern of the nasal mucosa in granulomatosis with polyangiitis

**DOI:** 10.1186/ar4041

**Published:** 2012-10-17

**Authors:** Janet Wohlers, Katrin Breucker, Rainer Podschun, Jürgen Hedderich, Peter Lamprecht, Petra Ambrosch, Martin Laudien

**Affiliations:** 1 University of Kiel, Department of Otorhinolaryngology, Head and Neck Surgery, Arnold-Heller-Straße 14, D-24105 Kiel, Germany; 2 Institute for Infection Medicine, University Hospital Schleswig-Holstein, Campus Kiel, Brunswiker Straße 4, D-24105 Kiel, Germany; 3 University of Kiel, Department of Medical Informatics and Statistics, Brunswiker Straße 10, D-24105 Kiel, Germany; 4 University of Lübeck, Department of Rheumatology, University Hospital of Schleswig-Holstein, Campus Lübeck, and Rheumaklinik Bad Bramstedt, Ratzeburger Allee 160, D-23538 Lübeck, Germany

## Abstract

**Introduction:**

In granulomatosis with polyangiitis (GPA), a complex autoimmune small-vessel vasculitis frequently associated with chronic necrotizing inflammation of the nasal mucosa, elevated nasal *Staphylococcus (S.) aureus *carrier rates are a risk factor for relapse. As cytokines are primarily involved in the regulation of defense against potentially pathogenic microorganisms, the aim of this study was to compare healthy individuals and GPA patients with respect to their baseline cytokine expression of nasal epithelial cells (NEC), which form the first barrier against such triggers. The ability of *S. aureus *to influence the nasal microenvironment's cytokine secretion was assessed by exemplary stimulation experiments.

**Methods:**

Baseline expression of 19 cytokines of primary NEC of GPA patients and normal controls (NC) was quantified by a multiplex cytokine assay. Stimulation experiments were performed with supernatants of *S. aureus *and expression of interleukin-8 was determined by ELISA.

**Results:**

In GPA, an altered pattern of baseline cytokine expression with significantly up-regulated G-CSF and reduced interleukin (IL)-8 concentrations was observed. Both NEC of GPA patients and NC responded to stimulation with *S. aureus*, but GPA patients displayed a significantly lower IL-8 secretion and a diminished dynamic range of response towards the stimulus.

**Conclusions:**

The data presented underline the hypothesis of a disturbed epithelial nasal barrier function in GPA. The dysregulated baseline expression of G-CSF and IL-8 and the reduced response to microbial stimulation may facilitate changes in the composition of the nasal flora and favour an imbalanced inflammatory response, which might be relevant for the disease course.

## Introduction

Granulomatosis with polyangiitis (GPA) is characterized by chronic necrotizing granulomatous inflammation with a predilection of the upper and lower respiratory tract and proteinase 3 (PR3) specific cytoplasmic anti-neutrophil cytoplasmatic antibodies (C-ANCA) [1,2].

So far, the pathogenetic mechanisms resulting in inflammation and autoimmunity in GPA are poorly elucidated. A complex interaction between genetic susceptibility and environmental factors is discussed [3-6], whereas low familiar clustering [7] stresses the importance of the latter.

Epidemiological studies revealed higher *Staphylococcus (S.) aureus *colonisation rates in GPA-patients compared to healthy and diseased controls [8-10]. Moreover, nasal carriage was shown to be associated with significantly elevated relapse rates, endonasal disease activity and first manifestation in the upper respiratory tract [8,11,12]. In addition, treatment with trimethoprim/sulfamethoxazole reduces the annual number of respiratory infections and the incidence of relapses in GPA-patients in remission [10,13,14].

Various staphylococcal superantigens were demonstrated to exert strong stimulatory effects on immunocompetent cells [15]. Furthermore, T-cells were shown to exhibit irregular modes of differentiation and cytokine expression upon stimulation with *S. aureus *[16,17], and *S. aureus *specificity was found in T-cell clones generated from peripheral blood lymphocytes of GPA-patients [17].

Remarkably, recent findings suggest a dysbalanced microbiom of the nasal cavity rather than a distinct microbial trigger comparable to the dysbiosis in inflammatory bowel diseases like Morbus Crohn [18]. Kain *et al*. demonstrated that in ANCA-associated systemic vasculitides highly prevalent auto-antibodies to lysosomal membrane protein-2 can cross-react with adhesins of gram-negative pathogens [19].

Another hint at microbial triggering of GPA is given by Pendergraft *et al*. demonstrating that patients harbouring PR3-ANCA also produce auto-antibodies to complementary PR3 (cPR3), a peptide translated from the antisense DNA strand of PR3. Conversely, the presence of cPR3 leads to production of both anti-cPR3 and anti-PR3 auto-antibodies. Genetic sequences that could be translated to cPR3-peptides were identified in numerous microbial and fungal organisms, including *S. aureus, Entamoeba histolytica *and Ross-River virus [20].

Taken together, the evidence is increasingly pointing to an imbalanced inflammatory response to microbial challenge as a potentially relevant process in the pathogenesis of GPA or *vice versa*.

Cytokines are significantly involved in the regulation of immune and inflammatory processes [21]. They enable inter-cellular communication and initiate immune responses by recruiting and activating specific immune cells, thus playing a decisive role in successful local defense against microorganisms [22]. Although aberrant cytokine patterns in GPA-patients in serum or plasma [23-25], mononuclear cells [26,27], pulmonary lesions [28,29] and bronchoalveolar lavage fluid [30] are verified by numerous studies, so far no study has been performed to assess altered cytokine expression in nasal epithelial cells, which form the first barrier against potential exogenous triggers.

We hypothesized an alteration in the prevailing pattern of cytokine expression of nasal epithelial cells especially focusing on microbial defense. The main objectives of this study were, therefore, to determine: i) the basal expression levels of 19 cytokines closely linked to microbial defense mechanisms on protein level in GPA-patients and normal controls and, furthermore, ii) the ability of *S. aureus *to influence the nasal microenvironment's cytokine secretion by exemplary stimulation experiments.

## Materials and methods

### Patients and biopsies

For analysing baseline cytokine secretion, nasal mucosa biopsies were obtained from 20 patients with GPA and 19 normal controls (NC). For stimulation experiments, biopsies of 10 GPA-patients and 10 NC were generated. Biopsies were taken from nasal turbinates at sites that were visually free of disease activity and suspect alterations like granuloma, edema, bloody patches, purulent secretion or crusts.

The study protocol was approved by the ethics committee of the University of Kiel, Germany (AZ A101/07) and was in accordance with the principles of the Declaration of Helsinki (latest revision October 2008). All patients provided written informed consent prior to enrolment. Exclusion criteria included pregnancy, haemostatic disorder and age of less than 18 years.

GPA was diagnosed according to the American College of Rheumatology classification criteria and Chapel Hill definitions for GPA as recommended by the European League Against Rheumatism (EULAR) [31]. GPA-subgroups were determined according to the European Vasculitis Study Group definitions and recent EULAR recommendations [31]. Extent of the disease was assessed by the Disease Extent Index (DEI) [32], disease activity was classified using the Birmingham Vasculitis Activity Score (BVAS) [33]. The Vasculitis Damage Index (VDI) was applied to record organ damage as a consequence of granulomatous inflammation and vasculitis [34,35]. All patients underwent a standardized interdisciplinary evaluation [36] and were examined endoscopically by an ear, nose and throat - specialist to evaluate endonasal GPA activity [2].

In order to assess the presence of systemic inflammation, white blood cell counts (WBC) as well as measurement of C-reactive protein (CRP) concentration and erythrocyte sedimentation rate (ESR) were performed. Besides slightly elevated ESR, as typical in GPA patients [37], all serologic parameters were within the normal range. In case of GPA, serologic titers of PR3-specific C-ANCA and myeloperoxidase-specific P-ANCA were determined according to Savige *et al. *[38].

In the normal control group, biopsies were taken while doctors were performing airway passage improving surgery. No individual of this group showed anamnestic or endoscopic signs of acute or chronic inflammatory or autoimmune alterations of the nasal mucosa or was under immunomodulating medication.

Detailed description of patient groups is given in Tables 1 and 2.

**Table 1:** Patients' characteristics

	Baseline cytokine secretion				Stimulation			
	**Normal control**		**Granulomatosis with polyangiitis**		**Normal control**		**Granulomatosis with polyangiitis**	
	**(n = 19)**		**(n = 20)**		**(n = 10)**		**(n = 10)**	

Age (yrs, mean and range)	39.50 (18 to 76)		51.55 (24 to 71)		43.30 (23 to 56)		50.80 (24 to 71)	
Sex	11 male, 8 female		12 male, 8 female		7 male, 3 female		7 male, 3 female	
First GPA manifestation till study entry (yrs, median and range)			3.8 (19 to 0)				3.3 (9 to 0)	
First GPA diagnosis till study entry (yrs, median and range)			1.6 (19 to 0)				3.1 (9 to 0)	

Serologic parameters								

	mean value	SD	mean value	SD	mean value	SD	mean value	SD
WBC (cells/nl)	6.74	1.55	8.22	2.82	7.87	1.97	7.77	2.33
ESR 1^st ^h (mm)	8.14	3.94	33.00	27.60	8.00	2.92	52.40	35.61
	median	range	median	range	median	range	median	range
CRP (mg/dl)	1.30	0.9 to 5.8	0.70	0 to 8.6	1.50	1.3 to 3.4	0.85	0.1 to 8.6
C-ANCA-titre (**1**:)			80	0 to 2560			640	0 to 2560
P-ANCA-titre (**1**:)			0	0 to 640			0	0 to 61

**Table 2:** Characteristics of patients with GPA

			Baseline cytokine secretion		Stimulation	
			n	%	n	%

**Biopsy proof**	GPA		12	60	7	70

**EULAR subgroups**	generalized		14	70	8	80
	early systemic		5	25	1	10
	local		1	5	0	0
	severe		0	0	1	10

**EULAR disease activity**	remission		6	30	2	20
	response		0	0	1	10
	relapse, minor		8	40	2	20
	relapse, major		4	20	3	30
	refractory		1	5	0	0
	low-activity		1	5	2	20

**Endoscopy: endonasal activity**	none		12	60	4	40
	mild		7	35	4	40
	moderate		0	0	2	20
	not evaluated		1	5	0	0

**Disease scores**			median (range)		median (range)	
	**DEI**		2 (0 to 5)		2 (0 to 5)	
	**BVAS-1**		3 (0 to 13)		1.5 (0 to 12)	
	**BVAS-2**		0 (0 to 4)		0 (0 to 4)	
	**VDI**		0.5 (0 to 3)		2 (0 to 4)	

**Immunomodulating therapy**			n (%)	mean value (mg)	n (%)	mean value (mg)
prednisolone			19 (95)	12.89	9 (90)	10.67
methotrexate			10 (50)	22.25	2 (20)	20
cyclophosphamide regular			3 (15)	133.33	1 (10)	100
cyclophosphamide bolus			2 (10)	1000	2 (20)	1000
azathioprine			2 (10)	125	2 (20)	125
leflunomide			2 (10)	25	2 (20)	25
mycophenolate mofetil			1 (5)	2000	0 (0)	0
cummulative cyclophosphamide			6 (30)	45.17 (g)	8 (80)	14.88 (g)

### Primary cell culture

Nasal epithelial cells (NEC) were enzymatically isolated from biopsies using dispase (Invitrogen, Karlsruhe, Germany). The cells were grown in Airway Epithelial Cell Growth medium (Promocell, Heidelberg, Germany) in a 96-well plate until pre-confluence with approximately equal numbers of cells per well were obtained. After a mean cultivation time of 13 days, supernatants were collected and stored at -80°C.

### Multiplex cytokine assay

The supernatants of NEC were analysed by performing a Bio-Plex™ Cytokine Assay according to the manufacturer's instructions (Bio-Rad Laboratories, Munich, Germany) allowing quantification of multiple cytokines with broad range standard curves in small-sample-sizes. Pro-inflammatory (interleukin (IL)-1α, IL-1β, IL-5, IL-6, IL-7, IL-8, IL-17, tumour necrosis factor-alpha (TNF-α)) and anti-inflammatory (IL-4, IL-10, IL-13) mediators, proteins mainly associated with adaptive immune responses (IL-2, IL-4, IL-12p70, IL-13, IFN-γ) or with recruitment of immune cells (IL-8, MCP-1, MIP-1), cytokines predominantly responsible for proliferation of inflammatory effector cells (G-CSF, GM-CSF) and control of apoptotic procedures associated with inflammation (TNF-β) were investigated.

Briefly, samples of 50 µl NEC supernatant were incubated with anti-cytokine antibody-coupled beads. After washing, the complexes were incubated with biotinylated anti-cytokine detection antibodies, and finally with streptavidin-phycoerythrin. Cytokine concentrations were measured using a Luminex 96-well plate reader (Bio-Plex™ 100-Multiplex-Suspension-Array-Reader; Bio-Rad Laboratories, Munich, Germany) and the Bio-Plex™ Manager Software 4.1.1 (Bio-Rad Laboratories, Munich, Germany). Human recombinant cytokines were used as standards.

### Stimulation of human nasal epithelial cells with S. aureus

For stimulation of NEC, supernatants containing the bacterial secretory products of *S. aureus *strain T190-2 (kindly provided by B.M. Bröker, University of Greifswald, Germany), which has been described to predominate in nasal isolates in Western Europe, was chosen. The *S. aureus *supernatants were not analysed for virulence factors secreted into the growth medium. However, in PCR analysis the test strain was positive for toxic shock and enterotoxin genes [39]. Experiments were performed corresponding to Sachse *et al. *[40]. According to the results of preliminary experiments concerning time and dose dependency (8, 12, 16, 24 hours; data not shown), stimulation experiments were performed with *S. aureus *supernatants diluted 1:5 in a final volume of 300 µl for 24 hours. An impact of the bacterial growth medium tryptic soy broth on the stimulation was ruled out before and NEC incubated in fresh cell culture medium without bacterial supernatants served as controls. Cell viability greater than 95% was verified by trypan blue dye exclusion test, and vital cell morphology was controlled in phase contrast microscopy. Moreover, 10 µl of cell culture supernatants were incubated on Columbia blood agar to prove absence of bacterial contamination. After stimulation, cell culture supernatants were collected and stored at -80°C until analysis.

### Quantification of IL-8 via ELISA

IL-8 concentrations in cell culture supernatants after stimulation were determined in duplicate by standard ELISA using the BD OptEIA human IL-8 set (BD Biosciences, San Diego, CA, USA).

### Statistical analysis

All statistics were performed in an exploratory manner using SPSS statistical software for Windows, version 18 (SPSS Inc., Chicago, IL, USA). Based on the Shapiro-Wilk-Test, normal distribution could be assumed for neither the entire Bio-Plex™ data nor for the delta values (stimulated - basal) of the stimulation experiments. Therefore, differences between the two groups (GPA versus NC) were evaluated by the nonparametric Mann-Whitney-U-Test. Normally distributed stimulation data were analysed by means of a general linear model for repeated measurement procedures in order to test the effects of within-subject factors (effect of stimulation within each group) and of between-subject factors (comparison of GPA- and NC-group). A *P*-value of ≤0.05 was regarded as statistically significant.

## Results

### Altered pattern of baseline cytokine expression in GPA-patients

For 17 of the analysed 19 cytokines in the supernatants of NEC, no difference between GPA-patients and normal controls could be detected (see Table 3 for details).

**Table 3:** Baseline cytokine expression of nasal epithelial cells (pg/ml)

	Normal control (NC)			Granulomatosis with polyangiitis (GPA)	NC vs. GPA
	median	range	median	range	*P*-value

**IL- 1α**	71.74	<1.51 to 319.24	92.38	7.90 to 477.11	0.322
**IL-1β**	<2.67	<2.67 to 8.67	2.70	<2.67 to 12.74	0.224
**IL-2**	<1.42	<1.42 to 6.46	3.60	<1.42 to 6.00	0.070
**IL-4**	<0.32	<0.32 to 0.80	<0.32	<0.32 to 0.72	0.283
**IL-5**	below detection limit (<2.51)				
**IL-6**	26.22	2.81 to 788.29	29.61	3.70 to 139.47	0.607
**IL-7**	below detection limit (<2.43)				
**IL-8**	1388.14	<1.77 to 5188.14	319.16	<1.77 to 3708.72	**0.009**
**IL-10**	below detection limit (<1.78)				
**IL-12**	<2.55	<2.55 to 3.75	<2.55	<2.55 to 4.03	0.283
**IL-13**	below detection limit (<2.68)				
**IL-17**	2.77	<1.87 to 6.41	3.54	<1.87 to 6.01	0.101
**G-CSF**	19.60	<1.31 to 204.41	61.30	1.53 to 663.58	**0.050**
**GM-CSF**	10.50	<0.74 to 28.70	12.50	7.74 to 21.56	0.084
**IFN-γ**	4.61	<1.61 to 21.41	5.92	2.27 to 18.18	0.089
**MCP-1**	3.66	<1.81 to 8.21	4.12	<1.81 to 7.90	0.667
**MIP-1β**	<1.41	<1.41 to 2.37	1.43	<1.41 to 2.79	0.141
**TNF-α**	<6.42	<6.42 to 13.46	<6.42	<6.42 to 11.00	0.296
**TNF-β**	9.44	<2.14 to 24.46	14.20	3.35 to 23.40	0.054

**Figure 1:**
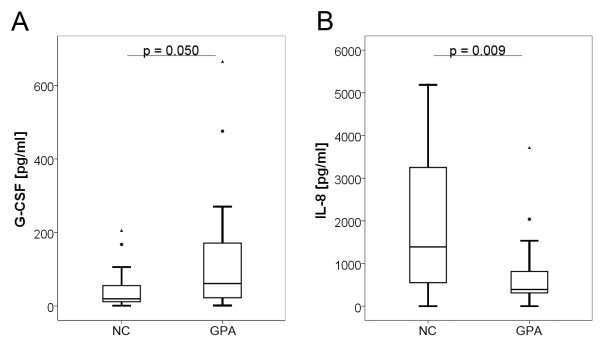
**Baseline cytokine expression**. Significantly altered baseline cytokine expression of primary nasal epithelial cells concerning G-CSF (**A**) and IL-8 (**B**) of granulomatosis with polyangiitis (GPA) patients compared to normal controls (NC). Data are represented as box plots showing the median as a straight line; the boxes correspond to the 75^th ^and 25^th ^percentiles and the whiskers indicate normal variation limits.

In contrast, NEC of GPA-patients showed significantly higher protein expression of granulocyte colony-stimulating factor (G-CSF, *P *= 0.050). Furthermore, concentrations of interleukin-8 (IL-8, CXCL8) were remarkably reduced in NEC of GPA-patients compared to NC (*P *= 0.009), as illustrated in Figure 1.

### Diminished response to stimulation with S. aureus culture supernatants in GPA-patients

Based on the results of the multiplex cytokine assay, a more detailed functional study of IL-8 expression of NEC upon stimulation with secretory products containing *S. aureus*-culture-supernatants was performed.

**Figure 2:**
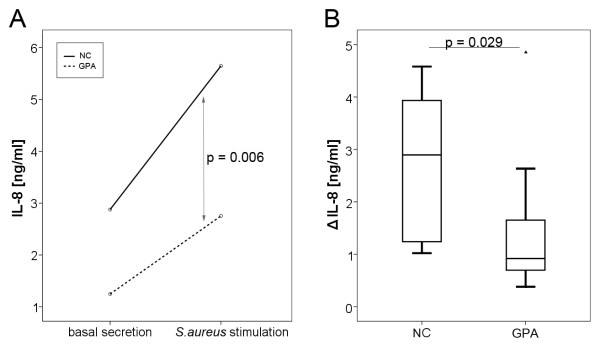
**Effect of stimulation with *S. aureus. ***Significant differences in IL-8 expression of primary nasal epithelial cells (NEC) comparing granulomatosis with polyangiitis (GPA) patients and normal controls (NC) before and after stimulation with *S. aureus *supernatants (**A**, *P *= 0.006). Furthermore, the dynamic range (Δ IL-8, secretion after stimulation minus basal secretion) is significantly reduced in GPA-patients (**B**, *P *= 0.029).

While both groups (GPA and NC) showed a statistically significant response to the stimulus (NC: *P *= 0.005, GPA: *P *= 0.005), baseline IL-8 expression (mean value 1,247.9, SD 881.6, maximum 2,735.3, minimum 379.0 pg/ml) as well as IL-8 expression after stimulation (mean value 2,753.0, SD 1,938.2, maximum 6,744.4, minimum 776.5 pg/ml) was significantly lower (*P *= 0.006) in GPA-patients compared to NC (basal: mean value 2,876.1, SD 1,498.3, maximum 4,935.5, minimum 500.0 pg/ml; stimulated: mean value 5,647.4, SD 2,407.9, maximum 9,516.1, minimum 1,521.9 pg/ml), as demonstrated in Figure 2A. Remarkably, also the dynamic range of the reaction (stimulated minus basal) was considerably more restricted in NEC of GPA-patients (*P *= 0.029, Figure 2B).

No correlation between nasal *S. aureus*-colonisation, endonasal GPA-activity or prednisolone dosages and these results could be detected (data not shown).

## Discussion

Cytokines' elaborate interplay enables inter-cellular communication and constitutes a crucial part in the regulation of immune and inflammatory processes - two important components in the pathogenesis of GPA - thus allowing an adequate response to microbial challenges.

Apart from Balding *et al., *who described an altered Th2-cytokine milieu in nasal biopsies [41], no comprehensive analysis of baseline cytokine expression in the nasal epithelium of GPA-patients has been performed so far.

For GPA, alterations in cytokine levels in pulmonary lesions have been described [28,29], but none of the 19 cytokines relevant for microbial defense included in this study were examined. Results of Richter *et al*., who reported elevated concentrations of the pro-inflammatory cytokines IL-1α, IL-1β and IL-6 in bronchoalveolar lavage fluid of GPA-patients [30], are difficult to compare to our findings obtained from a distinct compartment but hint at an altered cytokine spectrum.

Once induced by an inflammatory stimulus, almost all tissues within the human body are capable of producing G-CSF, thus leading to an increase in number and activation of neutrophils [42]. G-CSF serum levels are markedly elevated in response to infection and usually fall in parallel with the recovery process [43], whereas they remain elevated and correlate with disease activity in chronic inflammatory conditions, such as rheumatoid arthritis and Behçet disease [44,45]. Locally elevated G-CSF concentrations have also been observed in inflammatory bowel disease [46]. These findings match our results of locally elevated concentrations in NEC reasonably.

A prolonged life time of neutrophils resulting from a delayed apoptosis induced by G-CSF [47] increases the possibility of being primed and expressing PR3 on the cell surface [23,48]. Further activation through binding of ANCA can result in damage and lysis of endothelial cells [49]. In endothelial cells, G-CSF has been shown to down-regulate lipopolysaccharide-induced IL-8 expression [50], which would be conceivable for epithelial cells as well. Besides, the possible ability of G-CSF to reduce neutrophil killing of *S. aureus *[51] could have tremendous negative impacts on an effective immune defense.

IL-8 chemoattracts polymorphonuclear neutrophils (PMN) and monocytes and further promotes their activation [52], thus creating and maintaining an inflammatory microenvironment at the site of infection.

Lamprecht *et al. *suggested a down-regulation of monocytic production of IL-8 during the course of GPA [26], which is in line with our findings. Stimulation of PMN with IL-8, especially after previous treatment with TNF-α, leads to PR3 translocation to the cell surface [53], thus providing the prerequisite for interactions with PR3-ANCA, which directly activates diverse inflammatory reactions in PMN [54]. Variation of IL-8 expression levels is fine-tuned by graduated activation of at least three signaling pathways: NF-κB, JNK (JUN-N terminal protein kinase) and p38-MPK (mitogen-activated protein kinase) [55]. In order to evaluate whether this complex interplay is operating effectively in GPA-patients, we stimulated NEC with culture supernatants of *S. aureus*, which apart from its particular role in the pathogenesis of GPA has been shown to be a potent inducer of IL-8 expression in nasal epithelial cells [56,57]. The imbalance of IL-8 with a reduced baseline expression and a diminished response to bacterial stimulus of GPA-NEC could reasonably lead to a shift in the microbial flora towards an overbalance of facultative pathogenic microorganisms.

These data, in addition to the observation of a severely impaired ciliar beat frequency [58] and a reduced production of the antimicrobial peptide human β-defensin 3 of NEC upon stimulation with *S. aureus *[59], as well as an imbalanced regulation of genes involved in epithelial barrier function [18], fortify the hypothesis of considering GPA a disease with a disturbed epithelial barrier function as was also discussed for other chronic inflammatory diseases with nasal involvement [60].

However, this study has its limitations. Patients with GPA received immunomodulating therapy, for which negative impact on cytokine expression has been described [61,62]. Differential expression of the investigated cytokines (most of them without differences between NC and GPA) argues for successful prevention of such effects by cultivating NEC for an average of 13 days prior to the investigation.

The fact that the pattern of cytokine expression of NEC can be influenced by external stimuli paves the way for local pharmacological interference. Shifting the basal cytokine balance towards a higher IL-8 level, for example, by applying recombinant human IL-8 [63] or non-pathogenic bacteria components [64] could be a future therapeutic option.

## Conclusions

Taken together, our data suggest a specifically altered pattern of baseline cytokine expression of the nasal epithelium in patients with GPA compared to NC. This can facilitate changes in the composition of microbial colonisation and favour an imbalanced inflammatory response to microbial challenge and thus disease exacerbation. Our findings of an aberrant response to *S. aureus *stimulation in patients with GPA further underline this hypothesis. It remains to be investigated whether our results exemplify a general alteration in the reactive cytokine response to external stimuli. Taken into account the results of all 19 examined cytokines, we assume pathophysiological relevance of IL-8 and G-CSF in GPA, which could have potential therapeutic implications.

## Abbreviations

BVAS: Birmingham Vasculitis Activity Score; C-ANCA: cytoplasmic anti-neutrophil cytoplasmatic antibodies; cPR3: complementary PR3; CRP: C-reactive protein; DEI: Disease Extent Index; ELISA: enzyme-linked immunosorbent assay; ESR: erythrocyte sedimentation rate; EULAR: European League Against Rheumatism; G-CSF: granulocyte colony-stimulating factor; GM-CSF: granulocyte macrophage colony-stimulating factor; GPA: Granulomatosis with polyangiitis; IFN-γ: interferon-γ; IL: interleukin; JNK: JUN-N terminal protein kinase; MAPK: mitogen-activated protein kinase; MCP-1: monocyte chemotactic protein-1; MIP-1: macrophage inflammatory protein-1; NC: normal controls; NEC: nasal epithelial cells; P-ANCA: perinuclear anti-neutrophil cytoplasmatic antibodies; PMN: polymorphonuclear neutrophils; PR3: proteinase 3; *S. aureus: Staphylococcus aureus*; TNF: tumor necrosis factor; VDI: Vasculitis Damage Index; WBC: white blood cell count.

## Competing interests

The authors declare that they have no competing interests.

## Authors' contributions

ML, RP, JH and PA designed and coordinated the study. JW carried out the experiments. ML, JW, KB and PL were involved in data acquisition and interpretation. JH, JW and ML performed the statistical analysis. JW, KB, PL, RP, JH, PA and ML drafted and revised the manuscript. All authors provided final approval of the submitted manuscript.
